# Partial Trisomy of Chromosome 13 with a Novel Translocation (8 ; 13) and Unique Clinical Presentation in a Palestinian Infant

**DOI:** 10.1155/2019/4561761

**Published:** 2019-02-26

**Authors:** Allam Abuhamda, Aymen Elsous, Fadel A. Sharif

**Affiliations:** ^1^MOH Senior Consultant Neonatologist, Gaza Strip's Neonatal Intensive Care Units, Gaza Strip, State of Palestine; ^2^Assistant Professor, Faculty of Health Profession, Israa University, Gaza Strip, State of Palestine; ^3^Unit of Planning and Policy Formulation, Ministry of Health, Gaza Strip, State of Palestine; ^4^Genetic Diagnosis Lab, Islamic University of Gaza, Gaza Strip, State of Palestine

## Abstract

Partial trisomy 13 is a rare syndrome that usually culminates in death within the first six months of the infant's life. We present a rare case of partial trisomy 13q with exclusive clinical manifestations. The full-term male baby was born by normal vaginal delivery, his birth weight was 3500 grams, and head circumference was 30 cm. He had dysmorphic features in the form of microcephaly, trigonocephaly, depressed nose bridge, hypotelorism, long philtrum, high arch palate, left-sided inguinal hernia, hydrocele, and laryngomalacia. He was operated for pyloric stenosis at the age of 28 days. He also had left-sided severe pelvic-ureteral junction stenosis which was repaired by nephrostomy followed by pyeloplasty. Furthermore, he had right-sided vesicoureteral reflux grade III, right-sided hydronephrosis, small ventricular septum defect, small atrial septum defect, left lung lower lobe sequestration, and craniosynostosis of metopic suture. The baby had global developmental delay and failure to thrive. Cytogenetic study showed a 46,XY, der(8)t(8;13)(p23;q14) karyotype, emphasizing a partial trisomy 13q syndrome with a concomitant partial monosomy in 8p. The baby passed away, in the intensive care unit, at the age of 7 months due to respiratory failure resulting from recurrent chest infections. This is the first reported case of a partial trisomy 13q associated with chromosome 8 with unique clinical presentations. Cytogenetic study for both parents is recommended in order to pinpoint the origin of the translocation and to provide the proper counseling for the family.

## 1. Introduction

Trisomy 13 (Patau syndrome) is categorized as a full trisomy due to chromosome 13 nondisjunction at meiosis I or II, or mosaic (due to mitotic nondisjunction) and partial trisomy due to translocations. An unaffected parent can carry a balanced translocation between chromosome 13 and another chromosome. Robertsonian translocations may involve two chromosomes 13 or chromosome 13 and another acrocentric (14, 15, 21, 22) [1]. In mosaic trisomy 13, part of cells contains 3 copies of chromosome 13 and the other part contains 2 copies of chromosome 13 [2]. Partial trisomy 13 rarely occurs *de novo*. It mostly occurs when one of the parents harbors a balanced translocation involving chromosome 13. Partial trisomy 13 is exemplified by dysmorphic features, mental retardation, and psychomotor retardation [3]. Our case was diagnosed as a partial trisomy 13q syndrome with what we believe the first to result from translocation between (8p;13q) in an infant with unique dysmorphic features and clinical presentations.

## 2. Case Presentation

**Figure 1:**
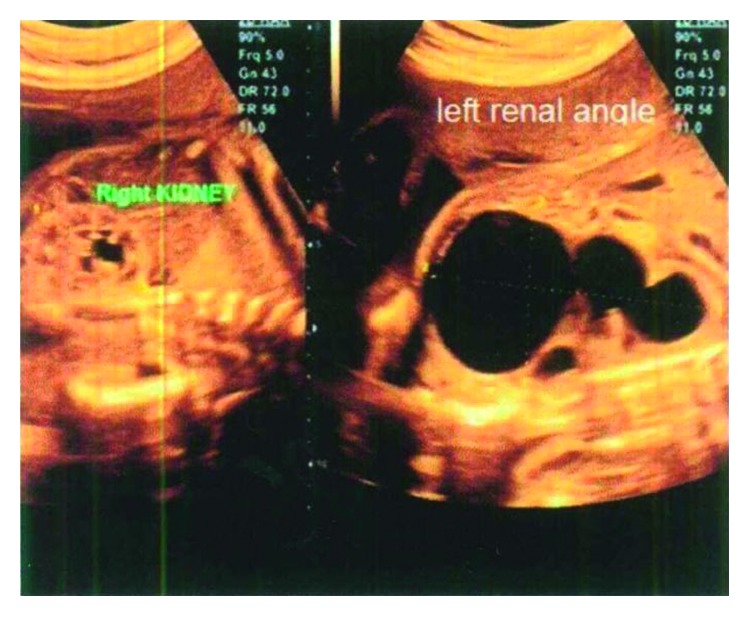
Antenatal ultrasound showed left-sided pelvic-ureteral junction stenosis.

**Figure 2:**
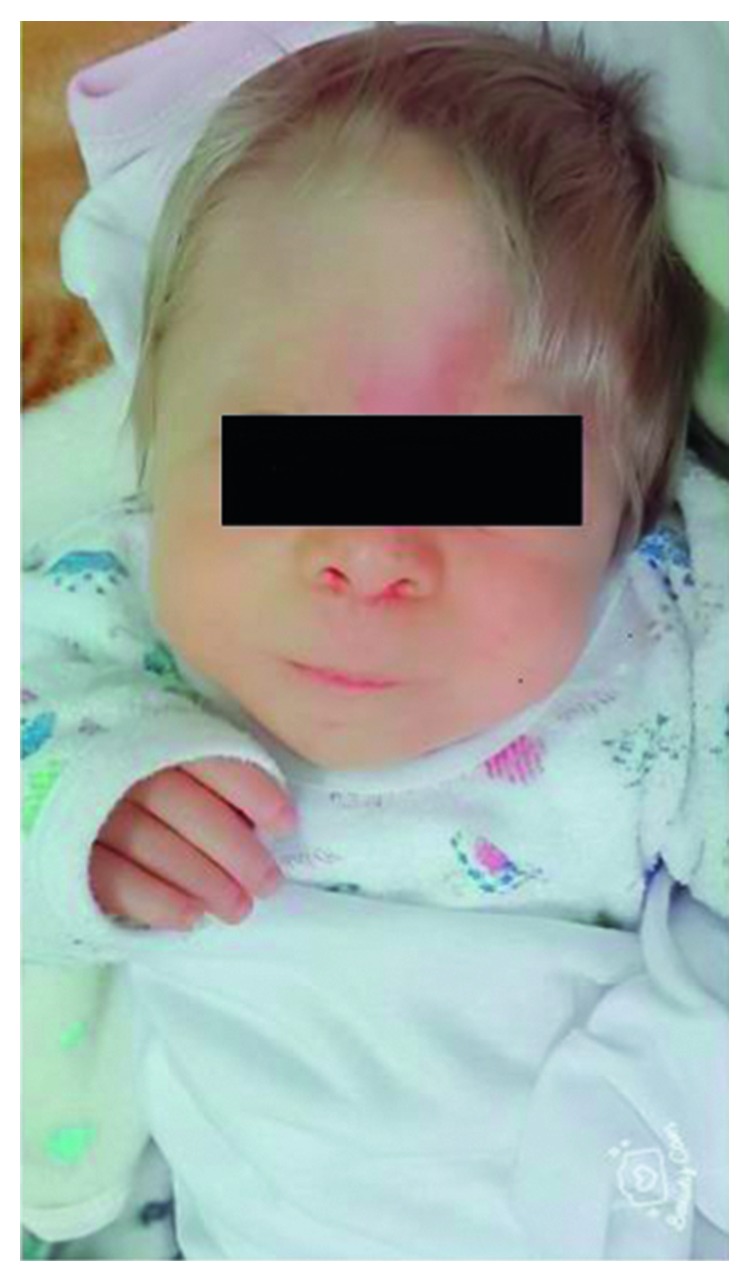
Trigonocephaly, hypotelorism, depressed nose bridge, and long philtrum.

The nonconsanguineous mother and father were both phenotypically normal and were 27 years and 34 years of age, respectively. They have two healthy daughters as well (5 and 4 years, respectively). None of the parents had a family history of congenital anomalies. The mother at 27 weeks gestational age did intrauterine ultrasound and showed severe left-sided pelvic-ureteral junction (PUJ) stenosis (Figure 1). The baby was born at term by normal vaginal delivery, and at birth, he presented with dysmorphic features in the form of microcephaly (head circumference 30 cm, below 3rd percentile), trigonocephaly, hypotelorism, depressed nose bridge, long philtrum, and high arch palate (Figure 2). The baby had ashy blonde hair, small flat hemangioma in the occipital area of the head, left-sided inguinal hernia, hydrocele, and laryngomalacia.

**Figure 3:**
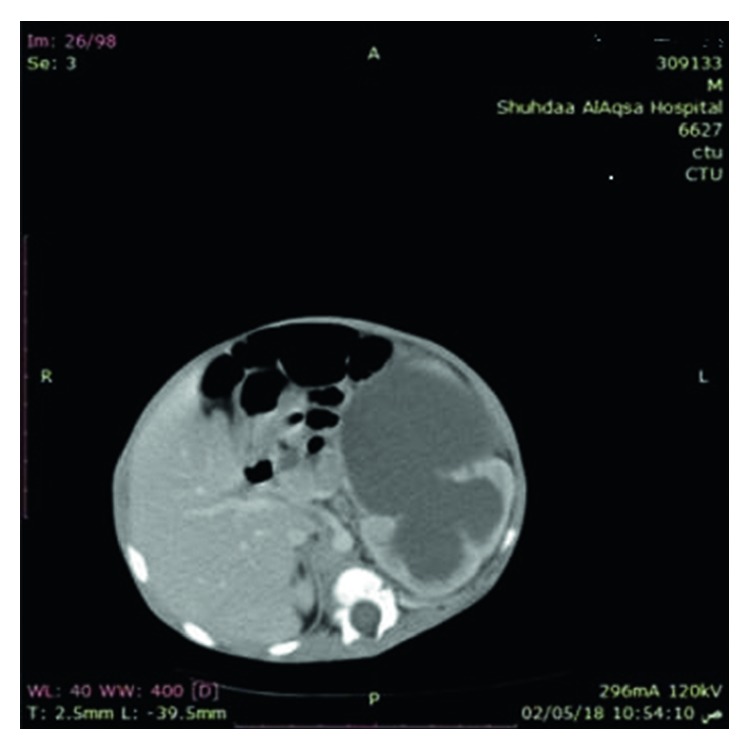
Abdominal CT showed left-sided pelvic-ureteral junction severe stenosis.

**Figure 4:**
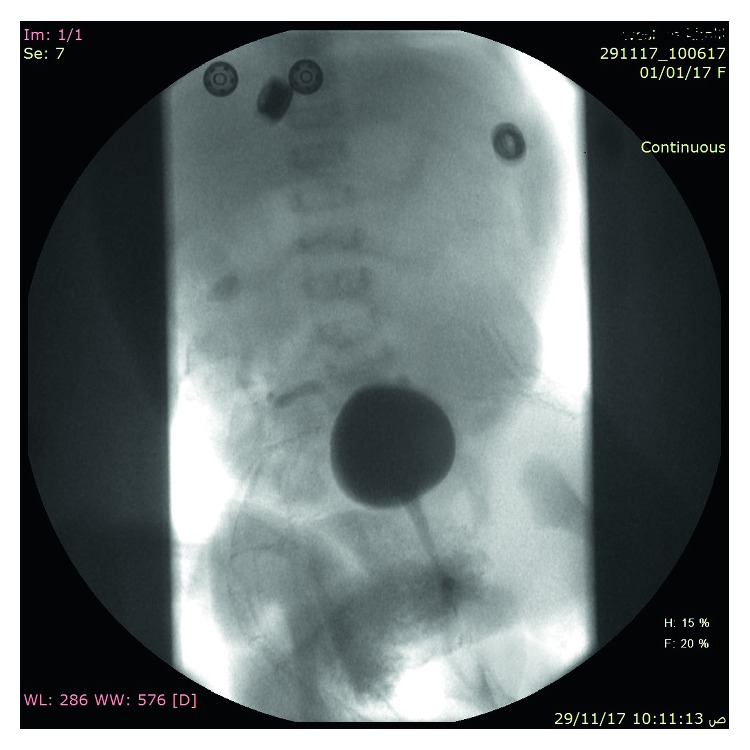
MCUG showed right-sided VUR grade III.

**Figure 5:**
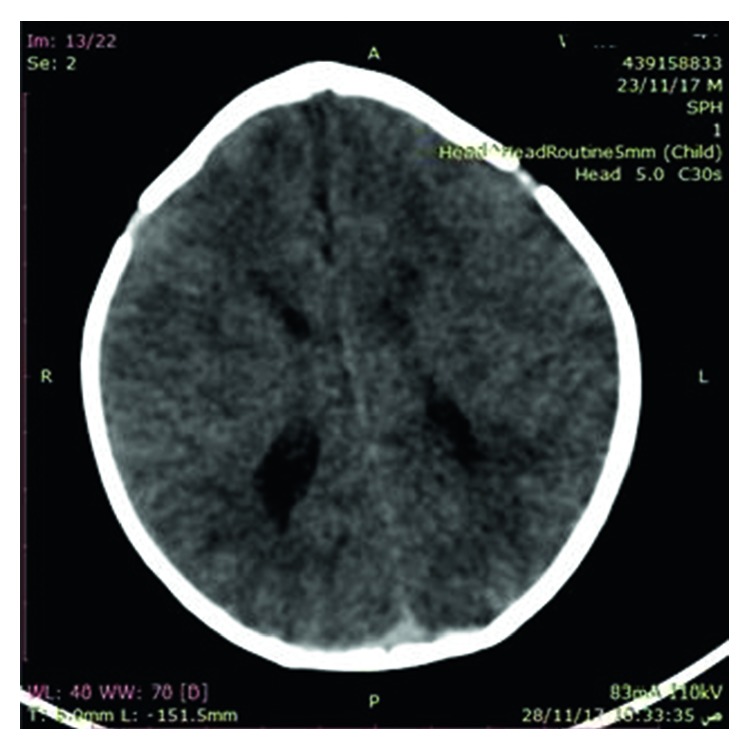
CT showed metopic suture craniosynostosis.

**Figure 6:**
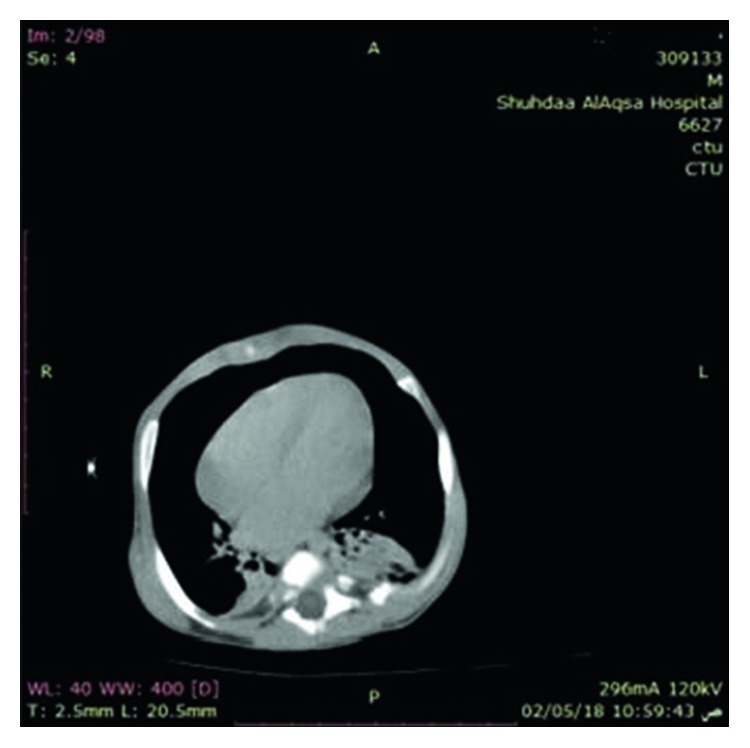
Chest CT showed the patchy area of consolidation in the posterior segment of the lower lobe of the lung.

Abdominal ultrasound and CT showed severe left-sided PUJ stenosis and right-sided hydronephrosis (Figure 3). MCUG showed right-sided vesicoureteral reflux (VUR) grade III (Figure 4). Left-sided nephrostomy was done and then operated at the age of 4 months for pyeloplasty. Urine output, serum electrolytes, and kidney functions were normal. The baby was operated at the age of one month for pyloric stenosis; then, he tolerated oral feeding and passed stool. Brain CT showed normal brain and craniosynostosis of metopic suture (Figure 5). By echocardiography, the baby had small atrial septum defect (ASD) and ventricular septum defect (VSD) with a left to right shunt. Chest CT showed patchy area of consolidation in the posterior segment of the lower lobe of the left lung which was highly suggestive of sequestration (Figure 6).

Chromosomes were prepared from the infant's peripheral blood using standard methods. Briefly, 0.5 ml heparinized blood was added to 10 ml complete RPMI-1640 in a sterile Falcon culture tube. For induction of lymphocyte proliferation, 2% (w/v) phytohemagglutinin-M was added to a culture tube. The tubes were incubated for 72 hours at 37°C under 5% CO_2_. Lymphocytes were arrested at metaphase by addition of colchicine (20 *μ*g/ml), 30 minutes before harvesting the cells. The cells were collected by centrifugation, resuspended in prewarmed (37°C) hypotonic solution (0.075 M KCl), fixed in methanol : acetic acid (3 : 1), and spread on thoroughly cleaned microscope slides. The preparations were aged at 80°C for 72 hours. Chromosome spreads were then banded by treatment with 0.25% trypsin for 3 to 10 seconds and staining in 4% Giemsa solution (pH 6.8) for 3 minutes. Twenty complete metaphase plates were visually analyzed, and karyotypes were prepared using a computerized Applied Spectral Imaging system. Karyotypes were interpreted according to the International System for Human Cytogenetic Nomenclature (ISCN, 2016) [4].

**Figure 7:**
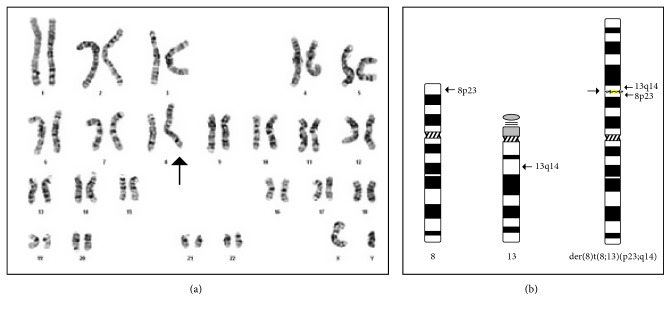
(a) Karyogram of the proband with derivative chromosome 8 (indicated by arrow) containing a large segment of the long arm of chromosome 13 resulting in unbalanced karyotype—partial trisomy 13q14-qter and partial monosomy 8p23-pter. (b) Ideograms of chromosome 8, chromosome 13, and der(8)t(8;13)(p23;q14); arrows indicate the breakpoints and the fusion in the derivative chromosome.

The karyotype of the infant revealed the presence of a derivative chromosome 8 where a major portion of the long arm of an extra chromosome 13 had attached to the telomeric end of the short arm of chromosome 8 (Figure 7). Therefore, the proband had partial trisomy for the major portion of the long arm of chromosome 13 (13q14 ⟶ qter) and partial monosomy for a small part of chromosome 8 (8p23 ⟶ pter). The infant karyotype can be formulated as 46,XY, der(8)t(8;13)(p23;q14). The parents were advised to consider karyotyping in order to find out the underlying cause of the translocation and inform them about the recurrence risk of the syndrome.

The baby, by his 7 month of age, was unable to roll over from back to stomach, unable to bring his hands to the middle line and putting toys in the mouth, and unable to babble but he had a social smile. He had a failure to thrive, at the age of seven months of age, and his weight was 5500 mg (below 5^th^ percentile) and his head circumference was 36 cm (below 5^th^ percentile). The baby had frequently been admitted to intensive care unit (ICU) ward due to recurrent chest infections that led to respiratory failure and death by the age of 7 months.

## 3. Discussion

The present infant had a partial trisomy 13q syndrome due to having a derivative chromosome: der(8)t(13;8)(q12;p23) not previously reported in the literature. It is expected that one of the parents (or both) is a carrier of a balanced translocation between the chromosomes (8;13). For this reason, chromosomal analysis of both parents is highly recommended to find out the origin of the derivative chromosome and provide the family with the proper counseling, such as recurrence risk and reproductive options [5]. However, the nonconsanguineous parents denied to undergo the karyotyping, probably to avoid blaming each other for the infant's disorder or to evade social stigma, especially if translocation is maternal in origin.

The constellation of the congenital anomalies observed in the present case can be largely attributed to the partial trisomy 13 [6]. However, the contribution of the missing portion of chromosome 8 (8p23 ⟶ 8pter) cannot be ignored. Isolated partial monosomy 8p23.1 has been linked to multiple manifestations, including prenatal and postnatal growth retardation, low birth weight, mild to moderate intellectual deficit, psychomotor retardation, and poor speech. Moreover, craniofacial abnormalities like microcephaly, high and narrow forehead, epicanthal folds, high arched palate, and low set ears. Furthermore, congenital heart defects (atrioventricular defects, septal defects, and pulmonary stenosis) and congenital diaphragmatic hernia [7, 8]. We believe that the partial trisomy 13q with the concomitant partial monosomy 8p is the cause of the multiple dysmorphic features and the novel clinical presentation, e.g., pyloric stenosis, pelvic-ureteral junction stenosis, hydronephrosis, lung sequestration, and the global developmental delay.

Exposure to teratogens can cause various structural abnormalities in the human chromosomes. In the last decade, Gaza strip has witnessed three consecutive wars in the years 2008, 2012, and 2014 in which different weapons including carcinogenic heavy metals have been used. Exposure to such heavy metals has been shown to cause double-strand breaks in the human DNA [9] and increase the incidence of congenital anomalies [10, 11].

## 4. Conclusion

The presented case is a partial trisomy 13q syndrome with 1st time occurrence of a translocation between chromosome parts 8p23 and 13q12 with exclusive dysmorphic features and clinical course. There was no past medical history for both parents which might indicate a *de novo* case. However, parental chromosomal analysis is recommended to rule out any chromosomal aberration. Meanwhile, exposure to mutagenic warfare heavy metals, as a predisposing factor, remains a source of speculation but should not be ignored because it was proved to have many mutagenic effects, including chromosomal anomalies.
